# Second and third year medical students’ self-reported alcohol and substance use, smoking habits and academic performance at a South African medical school

**DOI:** 10.4102/hsag.v24i0.1041

**Published:** 2019-09-23

**Authors:** Annelize Vorster, Anthonie M. Gerber, Lynette J. van der Merwe, Sanet van Zyl

**Affiliations:** 1 Department of Basic Medical Sciences, Faculty of Health Sciences, University of the Free State, Bloemfontein, South Africa; 2 School of Medicine, University of the Free State, Bloemfontein, South Africa

**Keywords:** Alcohol, Drug abuse, Medical students, Smoking habits, Academic performance

## Abstract

**Background:**

Health professional students frequently use alcohol and narcotics. The potential impact on academic performance and professional behaviour is concerning.

**Aim:**

This study aimed to determine self-reported use of alcohol, illicit substances (e.g. cannabis, lysergic acid diethylamide [LSD], magic mushroom, cocaine, crack, ecstasy, methamphetamine and heroin), prescription medication and smoking habits, correlating academic performance.

**Setting:**

Faculty of Health Sciences, University of the Free State.

**Methods:**

An observational, descriptive, cross-sectional study design was used. Information was obtained using a self-administered questionnaire, capturing demographics, self-reported academic performance, drinking and smoking habits, and substance use. Coded responses were analysed using the Remark Office OMR 8 Software System. Descriptive statistics were calculated for categorical variables.

**Results:**

Completed questionnaires comprised 171 students. A total of 78.4% of second year and 82.8% of third year students reported using alcohol. Twenty-two per cent of second year and 24.1% of third year students reported cannabis use. In the second year group, three (2.7%) students reported using magic mushroom, two (1.8%) reported cocaine, two (1.8%) reported ecstasy and one (0.9%) reported using methamphetamine. Only third year students reported using LSD or ‘crack’. Cigarette smoking was common – 31.5% and 35.1% in both groups, respectively. Smokeless tobacco devices were used by 8.5% of second year and 7.1% of third year students. Almost 40% of both groups reported that they had smoked a water pipe. Academic performance achieved was mostly 60% – 69% (38.9%) among second year students and 70% – 79% (46.6%) among third year students.

**Conclusion:**

Self-reported use of alcohol and drugs and smoking among medical students is alarming. Additional student support, early identification and referral for management and/or rehabilitation should be a priority at tertiary institutions responsible for training future healthcare professionals.

## Introduction

### Background

Alcohol abuse among students nationally and internationally is rising and alcohol has generally been identified as the most commonly used substance among students (SA Statistics 2013; Van Zyl et al. 2015). Hazardous drinking is defined by the volumes of alcohol consumed and/or the pattern of alcohol consumption associated with an increased risk for alcohol-attributable diseases in the future (Seggie 2012). Hazardous drinking has to be differentiated from alcohol use disorders, which have their own specific diagnostic criteria (Halme et al. 2008). According to data published by the National Institute on Alcohol Abuse and Alcoholism (NIAAA) (n.d.) in the USA, 80% of students drink alcohol. These results indicated that half of the students engage in binge drinking, where men consume five drinks and women consume four drinks in the course of 2 h.

Peltzer, Davids and Njuho (2011) have indicated that young adults have the highest levels of binge drinking and harmful drinking patterns in South Africa. O’Flynn (2011) defines harmful drinking where health problems develop because of alcohol consumption. This may include physical and/or psychological harm. A recent study by Van Zyl et al. (2015) at the University of the Free State focusing on alcohol consumption among all undergraduate students in residences found that 15.6% of students partake in hazardous drinking, 4.1% of students partake in harmful drinking and 5.6% of students are alcohol dependent. This is in line with data reported by international studies (Johnston et al. 2015).

Drug use among South African students and young adults is also alarmingly high (SA Statistics 2013; Van Zyl et al. 2015). According to SA Statistics (2013), at least 7.06% of the South African student population reportedly use some form of narcotic. A study conducted by Choi et al. (2011) at Weill Cornell Medical College in both New York and Qatar found that more than one-third of medical students reported excessive drinking and 5.0% reported non-prescription steroid use. The literature suggests that medical students abuse prescription drugs more commonly in comparison to students in general. This statement has, however, not been substantiated scientifically. International studies have found that lifetime use of both cocaine and amphetamines, used as tranquilisers during the last 30 days, occurred at considerably higher rates among medical students (Mesquita, Laranjeira & Dunn 1997; Van Zyl et al. 2015). Students have the perception that by using psychostimulants, they will enhance their concentration and ability to study (Bucher, Vu & Hojat 2013; Habibzadeh et al. 2011), even when it has not been prescribed to them by a medical practitioner, for example, in the case of attention deficit/hyperactivity disorder (ADHD). Medical students involved in previous studies have ‘justified’ their use of drugs, claiming that their scientific knowledge of the effects of illegal substances protect them from developing drug-related problems (Borsari & Carey 2001; Mesquita et al. 1997).

The driving factors for students’ drinking behaviour and substance use include peer pressure, social activities and external factors (Levy & Earleywine 2003; Van Heerden et al. 2009). In a study conducted among medical students in Spain, Mesquita et al. (1997) identified competitiveness, heavy workloads, contact with patients and the proximity of the residency examination as contributing factors to drug use. The emotional toll and responsibility when dealing with patients as part of daily routine training and as part of their clinical examinations cause students to rely on drugs in order to cope under these circumstances. This was also found in a South African study conducted by Gerber, Botes and Vorster (2016).

### Aim of the study

Data regarding alcohol and substance use and its effects on academic performance among medical students in South Africa are lacking. This study aimed to determine self-reported alcohol and smoking habits and substance use as well as self-reported academic performance in second and third year medical students at a South African medical school.

## Research methods and design

This was an observational, descriptive, cross-sectional study. It forms part of a larger study in a well-defined population-based sample of adolescents and young adults in the Faculty of Health Sciences, University of the Free State (UFS). The target population included all second and third year undergraduate medical students, aged 18 years and older, registered in the Faculty’s medical (MBChB) programme. These students are all involved in structured lectures presented by the School of Medicine and were willing to participate in a research project. First year students were excluded from this specific project in order to rule out possible other reasons or causes for using alcohol, other illicit drugs and/or smoking which might be specific to first year students only, for example, adjusting to a new social environment or adapting to the academic load and responsibilities. Medical students in their clinical years are again exposed to different daily responsibilities and stressors in comparison to the preclinical years of studying. They were therefore not included in this study, but might be included in similar future studies.

Students were approached after a lecture. The principal investigators organised data collecting sessions with the presenting lecturer involved and were present at all times. The necessary information pertaining to the study and information regarding participation was given to the students at each of these sessions, whereafter they were free to choose to participate or not. Questionnaires were then handed to the participating students to complete individually at their own pace. Participation was voluntary and questionnaires were completed anonymously. Completed questionnaires were collected and kept safe for data collection and analyses by the investigators.

The questionnaire used in this study was compiled by using information from various internationally validated questionnaires, with Cronbach’s alpha scores ranging between 0.74 and 0.90 (Bucholz et al. 1994; Center for Adolescent Substance Abuse Research 2009; Ialomiteanu & Adlaf 2015; Mayer & Filstead 1979; Wechsler et al. 1994). Careful attention was given to questionnaire design and questions applicable to South African students were grouped together. An initial group of 78 students voluntarily completed the questionnaire anonymously. Reviewing responses, results and data from this group, the questionnaire was deemed suitable for data collection as part of this study.

For the purpose of this study, information regarding demographic information, self-reported academic performance, drinking and smoking habits, and use of substances was obtained.

### Data analysis

Responses from the questionnaires were coded. The investigators captured and analysed the data from the completed questionnaires using the Remark Office OMR 8 Software System. Descriptive statistics, including frequencies and percentages for the categorical variables, were calculated.

### Ethical consideration

Approval to conduct this research was obtained from the Health Sciences Research Ethics Committee of the Faculty of Health Sciences at the University of the Free State (ETOFS 243/15). This includes authorisation from the responsible and applicable academic, research and student bodies involved. Participants received an information leaflet and the principal investigators explained the research objectives to them. Confidentiality was assured as no identifying data were recorded. The participants gave written consent before questionnaires were handed out for completion during a scheduled contact session.

## Results

### Demographic information

A total of 171 students participated in the study. The response rate was 96.6% (113/117) for second year and 46.8% (58/124) for third year students. In the second year, participants were mostly females (*n* = 70, 62.0%), whereas in the third year half of the participants were females (*n* = 29, 50.0%).The age range for the second year students was between 19 and 26 years (77.0% in the age group of 19–20 years).The age range for the third year students was between 19 and 25 years (86.2% in the age group of 20–22 years).

### Use of prescription medication and other illicit drugs

#### Medication for attention deficit/hyperactivity disorder

As shown in Table 1, 10 participants in both the second (9.3%) and third (17.2%) year groups, respectively, used prescribed medication for ADHD, while 12 (10.7%) in the second year group and 10 (17.2%) in the third year group used medication for ADHD without a prescription.

**TABLE 1:** Prevalence of the use of prescription medication among second and third year medical students at the University of the Free State over a period of 12 months.

Medication	Number of occurrences over a period of 12 months													
	1–2		3–5		6–10		11–19		20–39		≥ 40		Total	
	*n*	%	*n*	%	*n*	%	*n*	%	*n*	%	*n*	%	*n*	%
**Drugs for ADHD with prescription**														
Second year (*n* = 108)	1	0.9	1	0.9	4	3.7	0	-	0	-	4	3.7	10	9.3
Third year (*n* = 58)	2	3.5	1	1.7	2	3.5	0	-	0	-	5	8.6	10	17.2
**Drugs for ADHD without prescription**														
Second year (*n* = 112)	9	8.0	1	0.9	1	0.9	0	-	1	0.9	0	-	12	10.7
Third year (*n* = 58)	3	5.2	2	3.5	2	3.5	1	1.7	2	3.5	0	-	10	17.2
**Sedatives or tranquillisers with prescription**														
Second year (*n* = 111)	2	1.8	0	-	1	0.9	1	0.9	0	-	2	1.8	6	5.4
Third year (*n* = 58)	4	6.9	1	1.7	0	-	0	-	0	-	3	5.2	8	13.8
**Sedatives or tranquillisers without prescription**														
Second year (*n* = 110)	6	5.5	2	1.8	0	-	0	-	0	-	1	0.9	9	8.2
Third year (*n* = 58)	2	3.5	1	1.7	0	-	0	-	0	-	1	1.7	4	6.9

ADHD, attention deficit/hyperactivity disorder.

#### Sedatives and tranquilisers

There were six (5.4%) and nine (8.2%) second year students who reported using sedatives and tranquilisers with and without a prescription, respectively, while eight (13.8%) and four (6.9%) third year students reported using sedatives and tranquilisers with and without a prescription, respectively. These results are summarised in Table 2.

**TABLE 2:** Prevalence of the use of other substances and illegal drugs among second and third year medical students at the University of the Free State over a period of 12 months.

Substances and illegal drugs	Number of occurrences over a period of 12 months													
	1–2		3–5		6–10		11–19		20–39		≥ 40		Total	
	*n*	%	*n*	%	*n*	%	*n*	%	*n*	%	*n*	%	*n*	%
**Glue and other solvents**														
Second year (*n* = 110)	3	2.7	0	-	0	-	0	-	0	-	0	-	3	2.7
Third year (*n* = 56)	0	-	1	1.8	0	-	0	-	0	-	0	-	1	1.8
**Synthetic cannabis (‘Spice’)**														
Second year (*n* = 112)	0	-	0	-	0	-	0	-	0	-	0	-	0	-
Third year (*n* = 58)	7	12.1	1	1.7	1	1.7	1	1.7	2	3.5	2	3.5	14	24.1
**Cannabis**														
Second year (*n* = 112)	13	11.6	6	5.4	2	1.8	3	2.7	0	-	1	0.9	25	22.3
Third year (*n* = 58)	7	12.1	1	1.7	1	1.7	1	1.7	2	3.5	2	3.5	14	24.1
**LSD**														
Second year (*n* = 113)	0	-	0	-	0	-	0	-	0	-	0	-	0	-
Third year (*n* = 57)	3	5.3	0	-	0	-	0	-	0	-	0	-	3	5.3
**Magic mushroom**														
Second year (*n* = 113)	3	2.7	0	-	0	-	0	-	0	-	0	-	3	2.7
Third year (*n* = 57)	1	1.8	0	-	0	-	0	-	0	-	0	-	1	1.8
**Cocaine**														
Second year (*n* = 113)	2	1.8	0	-	0	-	0	-	0	-	0	-	2	1.8
Third year (*n* = 58)	2	3.5	1	1.7	0	-	0	-	0	-	0	-	3	5.2
**‘Crack’**														
Second year (*n* = 113)	0	-	0	-	0	-	0	-	0	-	0	-	0	-
Third year (*n* = 58)	2	3.5	0	-	0	-	0	-	0	-	0	-	2	3.5
**Ecstasy**														
Second year (*n* = 112)	1	0.9	0	-	1	0.9	0	-	0	-	0	-	2	1.8
Third year (*n* = 58)	1	1.7	0	-	0	-	0	-	0	-	0	-	1	1.7
**Methamphetamine**														
Second year (*n* = 113)	1	0.9	0	-	0	-	0	-	0	-	0	-	1	0.9
Third year (*n* = 58)	2	3.5	0	-	0	-	0	-	0	-	0	-	2	3.5
**Heroin**														
Second year (*n* = 113)	1	0.9	0	-	0	-	0	-	0	-	0	-	1	0.9
Third year (*n* = 58)	0	-	0	-	0	-	0	-	0	-	0	-	0	-
**Benzodiazepines**														
Second year (*n* = 113)	1	0.9	0	-	0	-	0	-	0	-	0	-	1	0.9
Third year (*n* = 58)	0	-	0	-	0	-	0	-	0	-	0	-	0	-

LSD, lysergic acid diethylamide.

#### Glue and other solvents

Three (2.7%) second year students and one (1.8%) third year student reported using glue and other solvents over a period of 12 months.

#### Synthetic cannabis and cannabis

None of the second year students reported using synthetic cannabis (‘spice’), while 14 (24.1%) third year students reported its use. Twenty-five (22.3%) second year and 14 (24.1%) third year students reported cannabis use. This included 11 (9.8%) second year and 5 (8.6%) third year students who reported using cannabis between 3 and 39 times over a period of 12 months, and 1 (0.9%) second year and 2 (3.5%) third year students who reported using cannabis more than 40 times over a period of 12 months.

#### Hardcore drugs

In the second year group, three (2.7%) students reported the use of magic mushroom, two (1.8%) reported cocaine, two (1.8%) reported ecstasy and one (0.9%) reported methamphetamine use. None of the second year students reported lysergic acid diethylamide (LSD) or ‘crack’ use. In the third year group, one (1.8%) student reported the use of magic mushroom, three (5.2%) reported the use of cocaine, one (1.8%) reported the use of ecstasy and two (3.5%) reported the use of methamphetamine. Three (5.2%) indicated the use of LSD and two (3.5%) reported the use of ‘crack’.

None of the second or third year students used ketamine, while one (0.9%) second year student reported heroine and benzodiazepine use, respectively, between one and two times during the past 1 year.

### Drinking habits

As shown in Table 3, a quarter of second year students (25.7%) had their first drink at the age of 18 years and 22.4% of the third year students started drinking at the age of 16 years. On a typical day, 20.4% of the second year and 25.9% of the third year students stated that they would drink between two and three alcohol-containing drinks. Almost one-third of the second year (*n* = 34, 30.1%) and 34.5% (*n* = 20) of the third year students bought their alcohol from a liquor store.

**TABLE 3:** Prevalence of the use of alcohol and related products among second and third year medical students at the University of the Free State over a period of 12 months.

Drinking habits	Number of occurrences over a period of 12 months													
	1–2		3–5		6–10		11–19		20–39		≥ 40		Total	
	*n*	%	*n*	%	*n*	%	*n*	%	*n*	%	*n*	%	*n*	%
**Alcohol**														
Second year (*n* = 111)	17	15.3	9	8.1	16	14.4	10	9.0	10	9.0	25	22.5	87	78.4
Third year (*n* = 58)	2	3.5	11	19.0	5	8.6	5	8.6	8	13.8	17	29.3	48	82.8
**Energy drink with alcohol**														
Second year (*n* = 110)	22	20.0	6	5.5	5	4.5	5	4.5	4	3.6	5	4.5	47	42.7
Third year (*n* = 57)	8	14.0	8	14.0	5	8.8	3	5.3	1	1.7	3	5.3	28	49.1
**Alcohol and cannabis**														
Second year (*n* = 113)	12	10.6	5	4.4	0	-	0	-	1	0.9	0	-	18	15.9
Third year (*n* = 58)	3	5.2	2	3.5	3	5.2	1	1.7	0	-	0	-	9	15.5

#### Alcohol

A total of 87 (78.4%) second year and 48 (82.8%) third year students reported the use of alcohol. In the second year group, 10 (9.0%) students reported using alcohol 20–39 times and 25 (22.5%) students reported using alcohol more than 40 times in the previous year. In the third year group, there were 8 (13.8%) students who reported using alcohol 20–39 times and 17 (29.3%) students who reported using alcohol more than 40 times in the previous year.

#### Combination of alcohol and energy drinks

The use of alcohol combined with an energy drink was reported by 47 (42.7%) second year and 28 (49.1%) third year students in the past 1 year. Nine (8.1%) second year students used an energy drink with alcohol between 20 and ≥ 40 times. Four (7.0%) third year students combined an energy drink with alcohol with a frequency of between 20 and ≥ 40 times.

#### Alcohol and cannabis

Eighteen (15.9%) second year and nine (15.5%) third year students reported using cannabis and alcohol together. Only one (0.9%) second year student reported using cannabis and alcohol together between 20 and 39 times over a period of 12 months, and none more than 40 times. None of the third year students reported using cannabis and alcohol more than 20 times over a period of 12 months.

### Smoking habits

The participants’ smoking habits are illustrated in Table 4. In both groups, almost one-third of students reported cigarette smoking over a period of 12 months. About 31.5% (*n* = 35) of second year and 35.1% (*n* = 20) of third year students reported cigarette smoking over a period of 12 months. Nine (8.5%) second year and four (7.1%) third year students reported using smokeless tobacco devices, and 45 (40.9%) second year and 23 (39.7%) third year students reported smoking a water pipe over a period of 12 months.

**TABLE 4:** Prevalence of the use of cigarettes and related products among second and third year medical students at the University of the Free State over a period of 12 months.

Smoking habits	Number of occurrences over a period of 12 months													
	1–2		3–5		6–10		11–19		20–39		≥ 40		Total	
	*n*	%	*n*	%	*n*	%	*n*	%	*n*	%	*n*	%	*n*	%
**Cigarettes**														
Second year (*n* = 111)	14	12.6	6	5.4	4	3.6	2	1.8	2	1.8	7	6.3	35	31.5
Third year (*n* = 57)	9	15.8	2	3.5	0	-	1	1.8	1	1.8	7	12.3	20	35.1
**Smokeless tobacco**														
Second year (*n* = 106)	4	3.8	2	1.9	3	2.8	0	-	0	-	0	-	9	8.5
Third year (*n* = 56)	2	3.6	1	1.8	1	1.8	0	-	0	-	0	-	4	7.1
**Water pipe**														
Second year (*n* = 110)	14	12.7	12	10.9	9	8.2	1	0.9	4	3.6	5	4.5	45	40.9
Third year (*n* = 58)	8	13.8	3	5.2	5	8.6	2	3.5	4	6.9	1	1.7	23	39.7

Fifteen (13.3%) second year students reported that they had started smoking over a period of 12 months compared to the nine (15.5%) participants in the third year group.

### Academic performance

Table 5 summarises the participants’ academic performance. One (0.9%) of the second year and none of the third year students reported average marks between 90% and 100%. In the second year group, 14.2% (*n* = 16) of the students reported average marks of 80% – 89%; 25.7% (*n* = 29) reported average marks of 70% – 79%, 38.9% (*n* = 44) reported average marks of 60% – 69% and 20.4% (*n* = 23) reported average marks of < 60%. As far as the third year group was concerned, 8.6% (*n* = 5) of the students reported average marks of 80% – 89%; 46.6% (*n* = 27) reported average marks of 70% – 79%, 41.4% (*n* = 24) reported average marks of 60% – 69% and 3.5% (*n* = 2) reported obtaining marks between 50% and 59%.

**TABLE 5:** Second and third year medical students’ current academic performance at the University of the Free State.

Year group	Current academic performance											
	90–100%		80–89%		70–79%		60–69%		50–59%		< 50%	
	*n*	%	*n*	%	*n*	%	*n*	%	*n*	%	*n*	%
Second year (*n* = 113)	1	0.9	16	14.2	29	25.7	44	38.9	20	17.7	3	2.7
Third year (*n* = 58)	0	-	5	8.6	27	46.6	24	41.4	2	3.5	0	-

According to students’ responses in the questionnaire, their self-perceived perception was that their social habits, including use of alcohol, drugs and/or smoking, did not negatively affect their academic performance.

## Discussion

This study confirmed that the self-reported prevalence of the use of alcohol and other substances of abuse, as well as smoking, among a group of undergraduate medical students is similar to findings in international literature. All types of drugs and alcohol are becoming more easily accessible and are used by populations much younger than it was in the past (Arora et al. 2016; NIAAA 2006).In 2013, the United Nations Office on Drugs and Crime reported that approximately 167–315 million people across the world between the ages of 15 and 64 years use illicit substances.

In this study, methylphenidate use with and without prescription was higher among third year students than among second year students. A previous study among medical students across all 5 years of the MBChB curriculum at the UFS reported that 9.8% of students used methylphenidate, and about one-third of these students were diagnosed with ADHD. Most students reported using stimulants to improve their ability to study as well as academic performance (Ialomiteanu & Adlaf 2015). Similar studies conducted at other South African medical schools found lifetime use of stimulants for non-medical purposes in approximately one-fifth of medical students, with only about 2% of these students diagnosed with ADHD (Jain et al. 2017; Retief & Verster 2016; Steyn 2016). These findings reported by Webb, Valasek and North (2013) are similar to the prevalence of methylphenidate use among medical students in the USA. Findings of a study conducted by Steyn (2016) at the University of Pretoria indicated a significant correlation (*p* < 0.001) between the use of methylphenidate and the rate at which alcohol, tobacco, cannabis, hard drugs and prescription medication were used.

The occurrence of drug use among the second and third year students was 7.2% and 12.2%, respectively. These students reported using drugs such as magic mushroom, cocaine, ecstasy and methamphetamine, while five (8.8%) third year students reported using LSD or ‘crack’. Four (2.3%) of the second and third year students also reported using glue and other solvents. Although these results are lower than figures reported in other international studies (Budhathoki et al. 2010; Drugwatch 2015), this reported risky behaviour in future healthcare professionals warrants intervention.

Alcohol use and abuse was seen to be very common in this group of students. The majority of second year (78.4%) and third year (82.8%) students reported using alcohol over a period of 12 months. Of concern was the reported alcohol use (more than 40 times over a period of 12 months) that was reported by 22.5% of second year and 29.3% of third year students. This is in line with international findings by Leinwand (2007), indicating an increase in the amount of alcohol consumed by students in general. In this group of students, 42.7% of second year and 49.1% of third year students reported using alcohol in combination with energy drinks, while 15.9% of second year and 15.5% of third year students reported a combination of alcohol and cannabis use.

Welcome et al. (2015) indicated that there are significant differences in academic performance among medical students who consume alcoholic beverages and those who totally abstain from it. In this study, although 78% – 83% of students reported using alcohol and 23% – 29% of students reported excessive alcohol use, students reported good academic performance. A total of 64.6% of second year and 87.9% of third year students reported obtaining marks between 60% and 79%. Less than one-fifth of second year and only two third year students reported obtaining marks below 60%. Alcohol use among the study participants does not seem to impact the academic performance as reported by students.

Fewer students reported smoking cigarettes than using alcohol, with approximately one-third of second and third year students reporting cigarette smoking. About 40% of second and third year students reported using a water pipe device, while less than 10% of second and third year students reported using smokeless tobacco devices. While cigarette smoking and tobacco use are detrimental to health, regular smoking is associated with other lifestyle choices and/or risky behaviours, such as drinking heavily, the use of cannabis and having multiple sexual partners (Emmons et al. 1998; Lejuez et al. 2003; Padrão et al. 2007; Rigotti, Lee & Wechsler 2000).

Almost one-fifth of all second year and almost a quarter of third year students in this study reported smoking cannabis. The health hazards of cannabis use include the effect of over-activation in cerebral areas, leading to altered sensory perception, changed sense of time, mood changes, impaired body movement, difficulty with problem solving and thinking, and impaired memory (National Institute on Drug Abuse n.d.). The long-term effects of cannabis include impaired learning and memory function.

Students have reported stress, such as stress associated with dealing with patients, as one of the important factors influencing their use of alcohol and other substances. This finding is supported by a study conducted by Arora et al. (2016) among a group of 230 undergraduate and postgraduate medical students in India, where 72.4% of participants reported psychological stress as the most common reason for using substances, followed by the need to reduce tiredness (46.8%) and peer pressure (42.6%).

Academic performance was not categorically rated as poor, average or high for the purpose of this study. Conclusions were drawn based upon whether changes in academic performance were noted by the individual students, based upon their use of alcohol or other substances. Although participating students mostly reported no adverse effects, that is, any negative impact from previous results, on their academic performance associated with their alcohol and substance use or cigarette smoking, the results indicate poor habits and lifestyle choices that could affect not only future academic performance but also patient care. This is supported by findings regarding capabilities and function among medical students indicating that academic stressors and working conditions are not ideal (Gerber et al. 2016).The rigours of medical training may place students at higher risk for burnout, depression, and low mental and emotional quality of life, which may lead to alcohol abuse (Jackson et al. 2016).

Based on the high prevalence of alcohol use and cannabis and cigarette smoking reported in this study, as well as small numbers of students reporting the use of ‘hardcore’ drugs, it would be valuable to implement awareness and intervention programmes for undergraduate medical students. Students in the second and third year of the 5-year curriculum at this institution have limited exposure to patients during preclinical training. The impact of increased responsibility associated with exposure to patients in the clinical training environment could contribute to exacerbation of the unhealthy, dangerous habits reported in this student group in later years of study. It is therefore imperative to address the issue of alcohol and drug abuse (and unhealthy lifestyle choices) among undergraduate medical students in order to ensure that they are able to function without impairment as future healthcare professionals.

### Study limitations

The response rate in the third year group was lower than expected because these students were preparing off campus for assessments at the time of completion of the questionnaires.

Surveys may be biased because participants must recall past experiences. The self-reported and sensitive nature of the information gathered in this questionnaire may have inhibited students and contributed to bias in findings, in spite of assurances of confidentiality and privacy.

Participants may not have responded fully in fear of potential repercussions. This could have resulted in under-representation of substance use. The small number of participants in this study limits the generalisability of the findings. It would be valuable to expand this study to other healthcare professions students at this institution, as well as to other institutions across South Africa.

## Conclusion

The high self-reported use of alcohol, drugs and cigarette and water pipe smoking among second and third year undergraduate medical students in this study is of great concern. Prevention, early identification and remediation of risky behaviour of future healthcare professionals should be an urgent priority.

### Recommendations

This study emphasises the need for follow-up studies to identify and address the driving factors responsible for the concerning prevalence of alcohol and drug use among medical students. Additionally, a holistic approach that includes prevention, early identification and emotional as well as social support programmes must be developed and incorporated in the curriculums of health sciences students.

The focus for alcohol and drug abuse prevention programmes should rather be on social and enhancement motives that resonate more with university students than emphasising the negative consequences of alcohol and drug use.
